# Indigenous Peruvians’ knowledge and awareness of dementia, risk and protective factors, and barriers to risk reduction

**DOI:** 10.1002/alz.095606

**Published:** 2025-01-09

**Authors:** Maritza Pintado Caipa, Laura Booi, Francesca R Farina

**Affiliations:** ^1^ Global Brain Health Institute, San Francisco, CA USA; ^2^ Global Brain Health Institute, Trinity College Dublin, Dublin, Ireland Ireland; ^3^ Leeds Beckett University, Leeds United Kingdom; ^4^ Global Brain Health Institute, Trinity College Dublin, Dublin Ireland

## Abstract

**Background:**

Emerging evidence suggests that Indigenous peoples may have disproportionately high rates of Alzheimer’s disease and related dementias (ADRD). However, there is a lack of data on Indigenous peoples’ knowledge and awareness of ADRD, especially in low‐ and middle‐income countries. Our aim was to investigate knowledge and awareness of ADRD, risk and protective factors, and barriers towards risk reduction in a remote Peruvian adult population.

**Method:**

Adults living in Amazonian, Andean highland, coastal Peruvian regions completed a pen and paper survey about dementia knowledge, risk and protective factors, and barriers to risk reduction. Data were analyzed using descriptive statistics.

**Result:**

Participants were 133 young adults (98 female; 1 non‐binary; mean age = 28.6 ± 6.4 years). Most participants identified as mixed race (i.e., Indigenous and European ancestry; 62.4%) or Quechua (28.6%). Six percent of the sample identified as White. Most participants lived in rural areas (60.2%). One third of participants were unemployed and half rated their socioeconomic status as low. Nearly one fifth of participants (18‐20%) reported not having electricity or a bathroom in their house, and one third (37.6%) did not have running water. Most participants (71.4%) said violence was a problem in their community. Half of participants (52.6%) said they were fearful about developing dementia. On average, participants recognized 5 of the 12 modifiable risk factors from the 2020 Lancet Commission on Dementia Prevention. The most endorsed factors were head injury (60.2%), social isolation (52.6%), and depression (51.1%). The least recognized factors were diet (9%) and air pollution (18%). The biggest barriers to risk reduction were lack of knowledge (63.9%), lack of money (55.6%), and pre‐existing health problems (49.6%).

**Conclusion:**

Peruvian adults living in remote areas have moderate knowledge dementia risk factors but identify multiple barriers to risk reduction. Findings are among the first to explore dementia knowledge in Amazonian, Andean highland and coastal regions of Peru.

